# Screw fixation in the treatment of displaced intra-articular calcaneus fractures: a systematic review protocol

**DOI:** 10.1186/s13643-022-02049-5

**Published:** 2022-09-11

**Authors:** Leah Wilmsen, Anne Neubert, Joachim Windolf, Andrea Icks, Bernd Richter, Simon Thelen

**Affiliations:** 1 Department of Orthopaedics and Traumatology, Medical Faculty and University Hospital Düsseldorf, Heinrich-Heine-University Düsseldorf, Düsseldorf, Germany; 2 TraumaEvidence @ German Society of Traumatology, Berlin, Germany; 3 Institute for Health Services Research and Health Economics, Centre for Health and Society, Medical Faculty and University Hospital Düsseldorf, Heinrich-Heine-University Düsseldorf, Düsseldorf, Germany; 4 Cochrane Metabolic and Endocrine Disorders Group, Institute of General Practice, Centre fpr Health and Society, Medical Faculty and University Hospital Düsseldorf, Heinrich-Heine-University Düsseldorf, Düsseldorf, Germany

**Keywords:** Calcaneus fracture, Calcaneal fracture, Systematic review, Screw fixation

## Abstract

**Background:**

The Calcaneus is the largest bone of the foot and the most frequent tarsal bone to be fractured. Overall, it causes round about 10 cases per 100,000 residents per year mainly in men. Especially displaced intra-articular calcaneus fractures often have early and late complications and its associated disability. There are various strategies for the treatment of displaced intra-articular calcaneus fractures, but the gold standard is still subject of a long-standing controversy. Minimally invasive procedures became more common in an attempt to reduce the high rate of complications associated with open reduction and internal fixation. With the increase in minimally invasive techniques, screw fixation also gained in significance. The current literature does not sufficiently elucidate whether the screw fixation is superior to other treatment options especially in relation to adverse events, health-related quality of life and postoperative pain. This study aims to investigate benefits and harms of treating displaced intra-articular calcaneus fractures (types II, III and IV according to Sanders) with screw fixation in adults.

**Methods:**

A systematic review will be conducted based on the principles described in the Cochrane Handbook. We will include adults with displaced intra-articular calcaneus fractures of Sanders type II, III and IV. The surgical method of screw fixation shall be compared to other surgical interventions to stabilise calcaneus fractures. Primary outcomes are serious adverse events, health-related quality of life and postoperative pain level. MEDLINE, CENTRAL, CINAHL, Web of Science and bibnet.org, ClinicalTrial.gov and the World Health Organization International Clinical Trials Registry Platform (ICTR) will be searched. Screening and data extraction will be performed by two authors independently. A third author will arbitrate disputes. Risk of Bias will be assessed with the Cochrane tool. Meta-analysis will be performed if participants, interventions, comparisons and outcomes are sufficiently similar to ensure a result that is clinically meaningful.

**Discussion:**

Due to the increasing use of minimally invasive techniques and the increasing use of screw fixation instead of open reduction and plate fixation, it is important to analyse the benefits and harms of screw fixation for calcaneus fractures. Screw fixation could, in the future, help to operate in a less invasive and tissue preserving manner while still achieving an adequate functional result for the patient

**Systematic review registration:**

CRD42021244695

**Supplementary Information:**

The online version contains supplementary material available at 10.1186/s13643-022-02049-5.

## Background

The calcaneus is the largest bone in the foot and the most frequent tarsal bone to be fractured. Overall, fractures of the calcaneus account for 2% of all fractures [1, 2]. However, most of the calcaneus fractures are intra-articular and the treatment is demanding especially due to the concomitant soft tissue injuries in up to 25% of cases [3, 4]. Calcaneus fractures often occur due to a high-energy trauma (such as car accidents) or a fall from high altitude (e.g. occupational accidents or attempted suicide) [1, 5]. This particular aetiology may explain the sex distribution, with males (30–50 years of age) nearly twice as often affected. The incidence is 10.5–12.5/100,000 per year in men and 3.8–6.3/100,000 per year in women [6–8]. Early and late complications can lead to long-term disability. The former can include neurovascular injuries, compartment syndrome and wound infections. The latter may include heel exostosis, tendinitis (e.g. peroneal tendon tendinitis), malunion and osteoarthritis [3].

Within the last 30 years various surgical strategies for the treatment of displaced intra-articular calcaneus fractures have been developed. Prior the calcaneus fracture was treatment non-surgical [4]. Whether this fracture is best treated with a surgical or a non-surgical method has been subject to a long-standing controversy. However, when it comes to displaced articular surfaces with the displacement (step-off or gap) greater than 2 mm, the surgical treatment is believed to achieve better functional outcomes than the non-surgical [9–13].

According to published studies and recommendations of the Arbeitsgemeinschaft für Osteosynthesefragen (AO) Vereinigung, the classical surgical treatment is in form of an open reduction and internal fixation (ORIF) using a fixed-angle plate [4, 12, 14]. The major adverse events with this type of surgical treatment is that it is highly invasive in a region of poor blood supply and thus high complication rates of up to 25% of cases are reported. Complications such as wound healing disorders, implant failure, deep infections, and further causes requiring revision surgeries are described in the literature [3, 4, 10, 15, 16]. Given the above, various minimally invasive procedures have been increasingly adopted in recent years. Apart from achieving similar functional outcomes, these procedures seem to have a lower risk of complications [17, 18].

With the increasing popularity of minimal invasive techniques also screw fixation gained in significance. Different types of screws and various insertion techniques were discussed and tested. These includes cannulated screws, non-cannulated screws and absorbable screw [19]. Screw fixation is believed to offer various advantages, such as minimally invasive insertion, without losing stability [17]. Further, screws are less rigid than a plate fixation and it is believed to be a suitable technique for patients with co-morbidities like diabetes. Nonetheless, some studies point out that the fracture fragments need to be big enough to hold the screws. Therefore, highly comminuted fractures will not be sufficiently repaired with a screw fixation [20]. Further, some publication point out that there is a concern for secondary loss of reduction with screw fixations [17]. Despite several systematic reviews exploring the various treatment option for displaced intra-articular calcaneus fractures the optimal treatment is still not elucidated [19, 21–24]. The current literature does not sufficiently answer whether the screw fixation is superior to other treatment options especially in relation to adverse events, health-related quality of life and postoperative pain.

### Objective

This study aims to investigate benefits and harms of treating displaced intra-articular calcaneus fractures (type II, III and IV according to Sanders) with screw fixation in adults. We attempt to compare various minimally invasive screw fixation techniques with other surgical procedures such as ORIF.

## Methods

This systematic review will be based on the Cochrane Handbook for Systematic Reviews of Interventions [25]. It is registered with PROSPERO (CRD42021244695). The protocol has been reported according to PRISMA-P [26] which is shown in Additional file 2.

### Eligibility criteria

#### PICOS scheme

Population: Adult patients with displaced intra-articular calcaneus fractures of type II, III and IV according to Sanders

Intervention: Screw osteosynthesis including cannulated and non-cannulated screws as well as absorbable and non-absorbable screws

Comparison: Other surgical procedures such as ORIF, k-wire and mini-plates

Outcome: Serious adverse events; health-related quality of life; postoperative pain levels; all other adverse events; co-morbidities and concomitant circumstances; functional outcomes; duration of surgery.

Study design: Randomised controlled trials (RCT), quasi RCTs and controlled clinical trials (CCTs)

Adults (skeletally mature, meaning at least 16 years old) with intra-articular fractures type II, III and IV according to Sanders will be included. Bilateral fractures will be accepted for inclusion since these fractures hardly differ from unilateral injuries [27]. Sanders type I fractures will be excluded as these do not pose an indication for surgery. Studies that included patients with severe medical impairments, such as a severe vascular or neurological disease, diabetes mellitus, or a known local or systemic infection will be excluded, since these are often associated with circulatory problems and, therefore, poor wound healing per se [28, 29]. All studies that describe polytrauma patients or severe additional injuries to the ipsilateral lower extremity as well as open fracture of the calcaneus will be excluded. This is because, when prioritising the treatment of a patient with multiple trauma, the treatment of calcaneus fractures has a lower priority, and the outcomes will differ from those fractures immediately treated. We will exclude all open fractures since these frequently occur in polytrauma patients where ipsilateral lower extremities are affected which makes the validation of the treatment of the calcaneus alone difficult [30].

We will compare various minimally invasive screw fixation techniques (using cannulated or non-cannulated screws, non-absorbable and absorbable screws) to other surgical procedures such as but not limited to ORIF, k-wire fixation and the use of the mini-plate. Studies with non-surgical treatments will be excluded.

### Information sources

The following electronic databases will be searched for suitable studies: MEDLINE via PubMed, Cochrane Central Register of Controlled Trials (CENTRAL), Cumulative Index to Nursing and Allied Health Literature (CINAHL), Web of Science via Science Citation Index and bibnet.org. The following clinical trial registries will also be searched: International Clinical Trials Registry Platform and ClinicalTrials.gov.

### Search strategy

The search strategy will be developed in consultation with an information specialist and subsequently adapted to each of the databases mentioned above to match the respective database’s characteristics. The search strategy includes the following keywords and their synonyms: calcaneus fracture(s), operative intervention(s) and screw fixation(s). The search will be limited in time to all studies published after 2000 since method of screw fixation was developed only in 2006. It will be limited to publications in German and English. A draft search strategy for a search on PubMed via MEDLINE is shown in Additional file 1.

## Study records

### Data management

The results from the literature search will be aggregated in Covidence software [31]. Further, the data extraction and information on risk of bias will be performed using the Covidence software. Thereafter, the data will be exported to the RevMan software which will facilitate the writing process in collaboration of all authors [32].

### Selection process

The trials will be selected by two review authors (LW and Adrian Deichsel (AD)), working independently of each other. The decision on whether to include or exclude a trial will be based on the inclusion and exclusion criteria as well as the PICOS. First, the review authors will analyse only titles and abstracts. Those selected will then be screened as full text. A third author (AN) will arbitrate in the event of a disagreement. The reasons for excluding a study in the stage of full text screening will be noted and reported.

### Data collection process

Two review authors (LW and AD) will use a previously developed form by the Cochrane Collaboration to extract data reported in the included studies [33]. First, the authors (LW and AD) will test the data extraction form on three included studies. If necessary, they will make changes to the form [34]. They will then extract the data from the included studies. In the event of a disagreement, a third author (AN) will be asked to arbitrate. In case of missing data or uncertainties, we will contact the corresponding trial author.

### Data items

We will extract data related to study characteristics (e.g. study design, setting, sample size), population characteristics (e.g. age, co-morbidities, Sanders type) included in the studies, intervention characteristics (e.g. type of screw fixation, approach used), comparators characteristics (type of surgery), outcomes described below including definitions and assessment tools used as well as additional information such as funding sources.

### Outcomes and prioritisation

#### Primary outcomes

The primary outcomes to be examined include serious adverse events, health-related quality of life and postoperative pain levels.

- Specific serious adverse events: for example, deep wound infections, compartment syndrome, or premature removal of metal implants—assessed by the physician; time of measurement: directly following the surgery (< 14 days postoperative) and after 3 months (> 12 weeks postoperative).
- Health-related quality of life: assessed using for example EQ-5D and SF-12 and rated by the patient; time of measurement: directly following the surgery (< 14 days postoperative), after 3 months (> 12 weeks postoperative) and after 1 year (> 1 year postoperative).
- Postoperative pain levels: determined using, e.g. the visual analogue scale or the numerical rating scale—rated by the patient. Time of measurement: directly following the surgery (< 14 days postoperative), after 3 months (> 12 weeks postoperative) and after 1 year (> 1 year postoperative).

#### Secondary outcomes

- All other adverse events, co-morbidities and concomitant circumstances, such as the occurrence of pulmonary embolism or myocardial infarction, as well as minor adverse events, such as superficial wound infections or minor screw loosening.
- Functional outcomes using foot-specific questionnaires, such as the American Orthopaedic Foot and Ankle Score (AOFAS), the Foot Function Index (FFI) and the Maryland Foot Score (MFS). In the context of questionnaires, radiological indices, such as Boehler’s and Gissane’s angles, which may provide information about future risk of osteoarthritis, should also be assessed. This is because the restoration’s quality (ideally, anatomical reduction) of the subtalar joint surface is the key predictive factor of future osteoarthritis. The questionnaires used contain patient-reported outcomes and physicians-rated sections.
- Duration of surgery, assessed in minutes per surgery

Secondary outcomes should be assessed at the same timepoints as the primary ones: directly following the surgery (< 14 days postoperative), when the fracture heals after 3 months (> 12 weeks postoperative) and after (> 1 year postoperative) to assess long-term effects.

### Risk of bias in individual studies

Two authors (LW and AD) will assess the risk of bias in each included study with the help of the Risk of Bias evaluation tool from Cochrane [35]. Emerging disputes should be clarified with the help of a third author (AN). To improve the reliability of the assessment, the instrument will be tested on three studies to ensure that the criteria for assessing the risk of bias are applied correctly and that a consensus can be established. The reasons for any differences of opinion should be investigated and resolved [36]. The study’s following characteristics will be evaluated: randomisation of study participants, allocation concealment, blinding of study participants and other parties, blinding of outcome assessment, handling of incomplete outcomes, selective reporting bias and other potential bias risks.

When assessing the risk of bias, a distinction is made between studies with a low risk of bias, unclear risk of bias and a high risk of bias. In low-risk studies, a low risk of bias is found across all domains. Studies are deemed to have an unclear risk of bias when not enough information, e.g. regarding the randomisation process is provided. A high risk of bias in at least one domain or across several domains is significantly reducing the confidence in the results of a study.

The main goal of risk of bias is to limit the meta-analysis to studies with a low risk of bias and evaluate the informative value of studies according to their risk of bias. This critical appraisal of the included studies should also assess their reliability, value and relevance of their outcomes. For this purpose, the study implementation, internal validity and external validity are considered [37]. Covidence will be used to enter data concerning the risk of bias.

## Data

### Synthesis

The overall goal is a quantitative synthesis of data from the included studies within a meta-analysis framework. A meta-analysis requires sufficient quality and homogeneity (clinical, methodological and statistical) of the included studies. Studies may differ methodologically and clinically from each other which could influence the degree to which the studies differ statistically. Statistical heterogeneity represents the random variation that is the basis of every study [38–40]. If the heterogeneity investigation reveals that the differences between the included studies are considerable, quantitative synthesis of data via meta-analysis will not be conducted. For the purposes of our systematic review, considerable heterogeneity shall be defined as *I*^2^ > 75%. This is true especially when the heterogeneity found cannot be explained by differences in methodology or clinical features of the included studies [41]. If no meta-analysis can be carried out, a qualitative analysis will be performed. The included studies will be summarised in tables and the results will be described narratively. If a meta-analysis is possible, we will use a random-effects model to determine the effect estimate. The results will be presented in a forest plot.

In case of missing data, the corresponding authors of the respective study will be contacted and asked to provide the missing data. If they do not respond, only the available data will be analysed. If standard deviations are missing, and there is no feedback from the contacted authors, these will if possible be calculated based on other available data (standard errors, 95% confidence intervals (CIs), exact *P* values). We will record and report each of these cases (missing data, calculated data, data provided by authors). Missing standard deviations will not be imputed. We shall examine the potential effects of the missing data within the meta-analysis.

The included studies are likely to exhibit significant methodological differences. When analysing the results, particular attention will be paid to how precisely randomisation was carried out. In particular, the number of observed cases should correspond to the number of randomised units. It should also be determined whether the separate individuals or the total number of fractures were considered. The unit of analysis, in this case, will be the calcaneus fracture as it is shown in studies that bilateral fractures occur [6]. Should there be an unusual large number of adjustments when including bilateral fractures, we shall carry out an additional sensitivity analysis. All data will be handled according to the protocol [42].

A sensitivity analysis will be performed to assess the impact of disputed decisions primarily. This analysis will be carried out for studies with a high risk of bias and studies with missing data. Such an analysis should also be carried out for the included RCTs and CCTs [42].

Subgroup analysis will be carried out to identify and examine potential effect modifiers. Such an analysis will be carried out for the different fracture types according to Sanders to determine whether the results of Sanders type II, III and IV differ from one another [42]. Moreover, the effect of age and gender should be analysed in more detail since males have a higher incidence.

### Meta-bias(es)

To counteract the distortion caused by publication bias as effectively as possible, we will search various databases for studies as described in section *Search strategy*. A funnel plot will be used to check for publication bias with the Eggers test for funnel plot asymmetry if at least ten studies are included in this systematic review. An asymmetric funnel plot could possibly indicate a reporting bias, chance or heterogeneity [43, 44]. If many small studies are included, which is likely to happen, the effect of the asymmetry of the funnel plot may be coated as small studies tend to selectively repress results more than larger studies. Furthermore, small studies are more prone for sampling error in their effect estimates [43–45].

### Confidence in cumulative evidence

We will present the overall certainty of the evidence for each outcome specified below, according to the GRADE approach, which takes into account issues related to internal validity (risk of bias, inconsistency, imprecision, publication bias) and external validity (such as directness of results). We will use GRADEpro GDT software [46] and justify all decisions to downgrade the certainty of the evidence by using footnotes.

We will present a summary of the evidence in a “Summary of findings” table. This will provide key information about the best estimate of the magnitude of effect, in relative terms and as absolute differences for each relevant comparison of alternative management strategies; the numbers of participants and studies addressing each important outcome; and a rating of overall confidence in effect estimates for each outcome.

We will report the following outcomes, listed according to priority:

1. Specific serious adverse events
2. Health-related quality of life
3. Postoperative pain
4. All other adverse events
5. Functional outcomes
6. Duration of surgery

## Discussion

Due to the increasing use of minimally invasive techniques such as screw fixation instead of open reduction and plate fixation, it is important to analyse the benefits and harms of screw fixation for the treatment of displaced intra-articular calcaneus fractures. Screw fixation could, in the future, help to operate in a less invasive and tissue preserving manner while still achieving good functional results with fewer adverse events for the patient. Our study will not be free of limitations. It is to be expected that most studies will only have small samples sizes. Unclear or high risk of bias may be present in the eligible studies due to issues with randomisation or blinding of personnel. We will assess the influence of high risk of bias studies by sensitivity analyses. In order to increase the amount of information, we will include quasi-RCT and CCT, even though these studies have no randomisation or an unclear randomisation process. We will attempt to evaluate the influence on the effect estimates by means of sensitivity analyses. However, it is possible that the included studies are of poor quality and/or considerable heterogeneity which will hinder a meta-analysis. In this case, we will attempt to analyse the included studies in qualitative manner to illustrate the results of the studies but also to discuss the limiting methodological issues within these studies. If changes to the protocol are necessary, these changes will be noted and rationales described.

## Supplementary Information

**Additional file 1.** Example: search strategy MEDLINE via PubMed.

**Additional file 2.** PRISMA-P 2015 Checklist.

## Data Availability

Not applicable.

## Abbreviations

AOFAS: American Orthopaedic Foot and Ankle Score
CCT: Controlled clinical trial
CI: Confidence interval
FFI: Foot Function Index
MFS: Maryland Foot Score
ORIF: Open reduction and internal fixation
PRISMA-P: Preferred Reporting Items for Systematic Reviews and Meta-Analysis for Protocols
Quasi-RCT: Quasi-randomised control trial
RCT: Randomised control trial
RoB: Risk of bias
