# Supplementary material for: Screw fixation in the treatment of displaced intra-articular calcaneus fractures: a systematic review protocol
Source: Syst Rev. 2022 Sep 11;11:199. doi: 10.1186/s13643-022-02049-5 (PMC9465885; doi:10.1186/s13643-022-02049-5)
Supplement: Supplementary file 1 — Additional file 1. Example: search strategy MEDLINE via PubMed. [file 13643_2022_2049_MOESM1_ESM.docx]

**Additional file 1**

Example: search strategy MEDLINE via PubMed:

1. "screw fixation"[All Fields] OR "percutaneous fixation"[All Fields] OR "minimally invasive"[All Fields] OR "calcium sulfate cement"[All Fields] OR "Bone Screws"[MeSH Terms]
2. "Calcaneus"[MeSH Terms] OR "calcaneus/injuries"[MeSH Terms] OR "calcaneus/surgery"[MeSH Terms] OR "calcaneus/therapy"[MeSH Terms] OR "Calcaneus"[MeSH Terms] OR "Calcaneus"[All Fields] OR "calcaneitis"[All Fields] OR "calcaneous"[All Fields] OR "Calcaneus"[MeSH Terms] OR "Calcaneus"[All Fields] OR "calcaneal"[All Fields]) OR "Calcaneus"[MeSH Terms] OR "Calcaneus"[All Fields] OR "calcaneum"[All Fields]
3. "Calcaneus"[MeSH Terms] OR "Calcaneus"[MeSH Terms] OR "Calcaneus"[All Fields] OR "calcaneitis"[All Fields] OR "calcaneous"[All Fields] OR "Calcaneus"[MeSH Terms] OR "Calcaneus"[All Fields] OR "calcaneal"[All Fields] OR "Calcaneus"[MeSH Terms] OR "Calcaneus"[All Fields] OR "calcaneum"[All Fields]
4. ((randomized controlled trial [pt] OR controlled clinical trial [pt] OR randomized [tiab] OR placebo [tiab] OR clinical trials as topic [mesh:noexp] OR randomly [tiab] OR trial [ti])) NOT (("animals"[MeSH Terms] NOT "humans"[MeSH Terms]))
5. #1 AND #2 AND #3 AND#4
